# *LIPH* Expression in Skin and Hair Follicles of Normal Coat and Rex Rabbits

**DOI:** 10.1371/journal.pone.0030073

**Published:** 2012-01-17

**Authors:** Mathieu Diribarne, Xavier Mata, Julie Rivière, Stéphan Bouet, Anne Vaiman, Jérôme Chapuis, Fabienne Reine, Renaud Fleurot, Gérard Auvinet, Séverine Deretz, Daniel Allain, Laurent Schibler, Edmond-Paul Cribiu, Gérard Guérin

**Affiliations:** 1 INRA, UMR1313, Unité de Génétique Animale et Biologie Intégrative, Jouy-en-Josas, France; 2 INRA, UR0892, Virologie et Immunologie Moléculaires, Jouy-en-Josas, France; 3 INRA, Plateforme de Microscopie et Imagerie des Micro-organismes, Jouy-en-Josas, France; 4 INRA, UR967, Génétique Expérimentale en Productions Animales, Surgères, France; 5 INRA, UR631, Station d'Amélioration Génétique des Animaux, Castanet Tolosan, France; Institut Jacques Monod, France

## Abstract

Natural mutations in the *LIPH* gene were shown to be responsible for hair growth defects in humans and for the rex short hair phenotype in rabbits. In this species, we identified a single nucleotide deletion in *LIPH* (1362delA) introducing a stop codon in the C-terminal region of the protein. We investigated the expression of *LIPH* between normal coat and rex rabbits during critical fetal stages of hair follicle genesis, in adults and during hair follicle cycles. Transcripts were three times less expressed in both fetal and adult stages of the rex rabbits than in normal rabbits. In addition, the hair growth cycle phases affected the regulation of the transcription level in the normal and mutant phenotypes differently. *LIPH* mRNA and protein levels were higher in the outer root sheath (ORS) than in the inner root sheath (IRS), with a very weak signal in the IRS of rex rabbits. *In vitro* transfection shows that the mutant protein has a reduced lipase activity compared to the wild type form. Our results contribute to the characterization of the *LIPH* mode of action and confirm the crucial role of *LIPH* in hair production.

## Introduction

**Figure 1:**
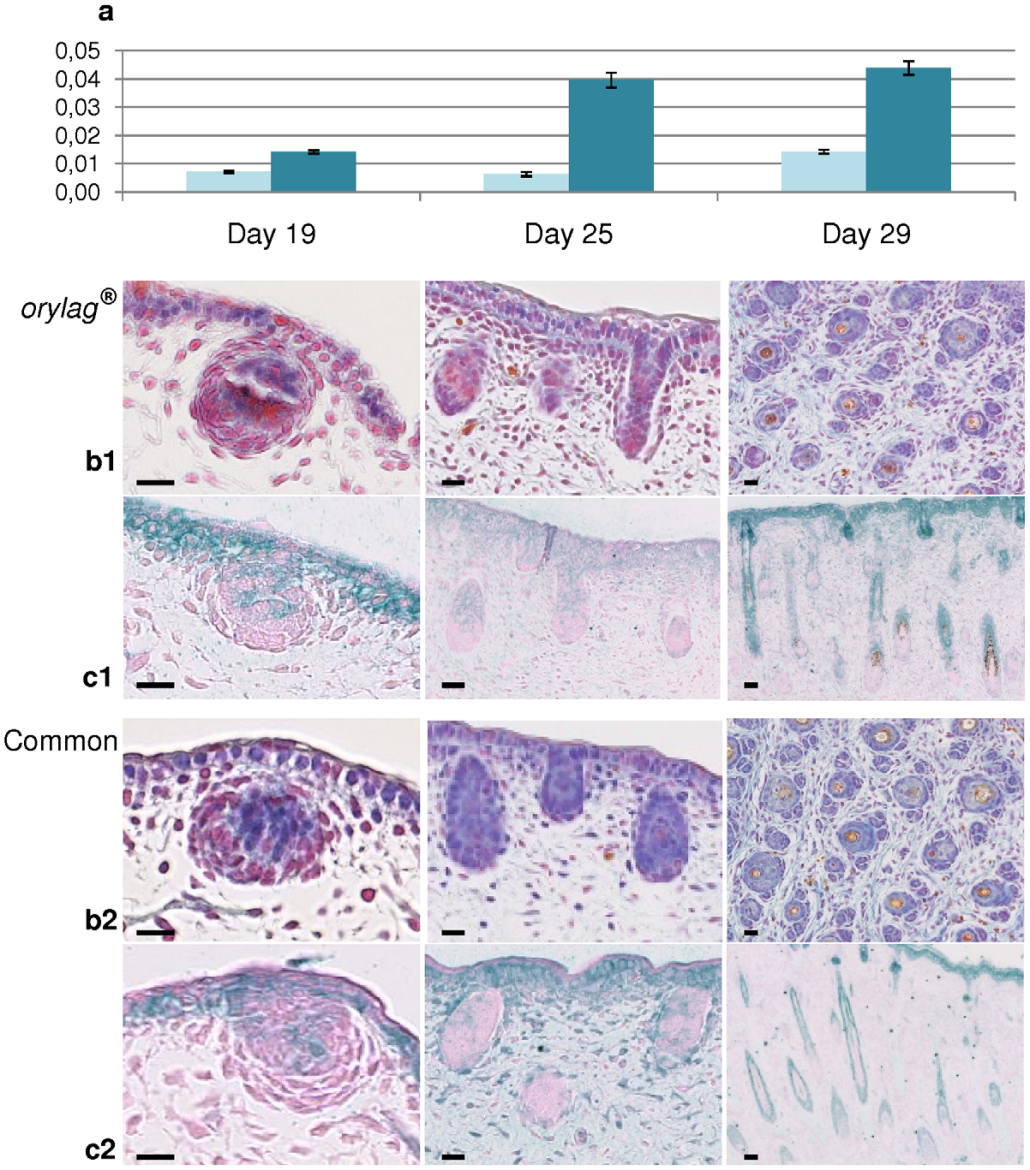
*LIPH* expression at three different fetal stages (days 19, 25 and 29) corresponding respectively to the formation of the central primary, lateral primary and secondary hair follicles. (Bars = 20 µm). (A) Q-PCR expression of *LIPH* mRNA in the *orylag®* rabbit skin (light blue bars) and in the normal rabbit skin (dark blue bars). The Y axis represents the relative expression level of *LIPH*. (B) Histology (ROAN staining) of *orylag®* (B1) and normal rabbit skins (B2). (C) Immunohistochemistry of *orylag®* (C1) and normal rabbit skins (C2).

In rabbits, hair follicles are structured into groups, usually constituted of one central primary hair follicle surrounded by 2–4 lateral primary hair follicles and by 20–50 secondary down hair follicles (Figure 1). These three types of hair follicles appear sequentially during fetal development and early after birth. At day 19 of gestation (the average gestation in rabbits lasts 30 days), the central primary hair follicles rise followed at day 25 by the primary lateral hair follicles. At day 29 of gestation, a secondary hair follicle for each of the 2 to 4 lateral hair follicles appears. Finally, secondary derived hair follicles emerging from the skin by the same hair channel, appears during the early childhood of the animals [1]. Normal rabbit fur is composed of three different types of hairs: guard hairs produced by central primary hair follicles (3–4 cm long for a diameter of 50–60 µm), awn hairs produced by lateral primary hair follicles (3–3.5 cm/25–30 µm) which both constitute the physical “outer coat” protection, and down hairs produced by secondary hair follicles (2.5–3 cm/15 µm), and the inner coat for thermal protection. Down hairs are the most abundant and represent about 90–95% of all hairs. In 1919, a mutant phenotype (rex) with soft short hairs was observed by a French breeder in a litter of European rabbits (*Oryctolagus cuniculus*) [2]. In the early nineteen-eighties, the rex trait (downs >95%) was improved by selection on an INRA experimental farm to further reduce the number of guard and awn hairs and is now commercialized as *orylag®* (downs >98% with a residual variability of downs percentage). The “r1” mutation was confirmed to segregate within INRA families as a monogenic, autosomal and recessive trait. Furthermore, a deletion of a single nucleotide in exon 9 of the *LIPH* gene (1362delA) was identified in rex rabbits [3]. This mutation results in a frameshift and introduces a premature stop codon shortening the predicted protein by 19 aminoacids. *LIPH* is a membrane-bound member of the mammalian triglyceride lipase family, the phosphatidic acid-selective phospholipase A1 (PLA1). It specifically hydrolyzes phosphatidic acid (PA) to produce 2-acyl lysophosphatidic acid (LPA), which is a lipid mediator with diverse biological properties including stimulation of cell proliferation and motility [4]. Disruption of the *LIPH* gene in the mouse results in various phenotypes including retarded hair growth and postnatal lethality [5]. In 2006, a deletion in the *LIPH* gene was identified as being responsible for Hypotrichosis Simplex (HS) in 50 families of Russian people [6]. Hairs of these patients are abnormally short, dystrophic and fragile due to retarded or arrested hair growth. Mutations have also been reported in other exons for HS and for Autosomal Recessive Woolly Hair syndrome (ARWH) partly associated to HS [7]–[14]. It is known that the *LIPH* gene is expressed abundantly in human HF as detected by RT-PCR without precise localization [6]. Later, *in situ* hybridization studies of skin sections showed that *LIPH* mRNA is strongly expressed in the human precortex, hair shaft cuticle and Huxley layer of the inner root sheath (IRS) of the bulb portion and in a more prominent manner in the outer root sheath (ORS) in the upper portion of the HF [12]. The mPA-PLA1 alpha (*LIPH*) protein was found to be associated with the detergent-resistant membrane fraction of insect transfected cells [15] confirmed by confocal fluorescence microscopy [16]. In spite of its important role in the hair growth process, little is known about the way *LIPH* is implicated in the hair follicle function and its expression, localization and exact role are still a subject of speculations. In this study we analyzed the spacio-temporal expression of the *LIPH* gene in fetal and adult rabbit skin by quantitative PCR as well as in hair follicles by *in situ* hybridization and immunochemistry. The expression of *LIPH* mRNA and protein was compared for normal and rex phenotypes. In addition, the activity of both normal and mutant proteins was estimated *in vitro* using transfected mammalian cell cultures.

## Results

### *LIPH* expression during fetal hair follicle development

The organization of the cell structures observed after Roan staining of the skin sections validated the expected timing of the formation of the different types of hair follicles (HF) in normal rabbits (Figure 1, row B2). At day 19, the bulb of the central primary hair follicles appeared in both phenotypes with a higher meiotic activity of the external cells revealed by the red coloration. At day 25, the epidermis was well organized and looked very much the same in normal and *orylag®* skins. The central primary HF was well visible as well as primary lateral HF in formation from the epidermis layers by invagination (Figure 1B1). It seemed that the meiotic activity was more intense in the parts that could be identified as the bulge and the bulb. At day 29, the group structure of the HF was visible with the apparition of the lateral primary HF in both phenotypes but with observable secondary HF in the normal rabbit not seen in the *orylag®*. Hair shafts were clearly identified in some of the central primary HF in both phenotypes (Figure 1, row B1). There was no apparent noticeable hair follicle group density difference between normal and *orylag®* rabbits. The mRNA expression level estimated by quantitative RT-PCR was significantly different at the three different stages of gestation between the *orylag®* and the normal rabbit skins (H = 9.9; p = 0.0017). There was on average three times less expression in the *orylag®* fetal skin than in normal fetal rabbit skin (Figure 1A). The presence of the m*PA-PLA1* was detected by immunochemistry at all fetal stages. At day 19, it was present in the epidermis and in the center of the growing central primary HF in both phenotypes. At day 25 and day 29, it seemed absent from the lower part of the follicle bulb in normal and in *orylag®* skin sections.

### *LIPH* expression in adult rabbits

**Figure 2:**
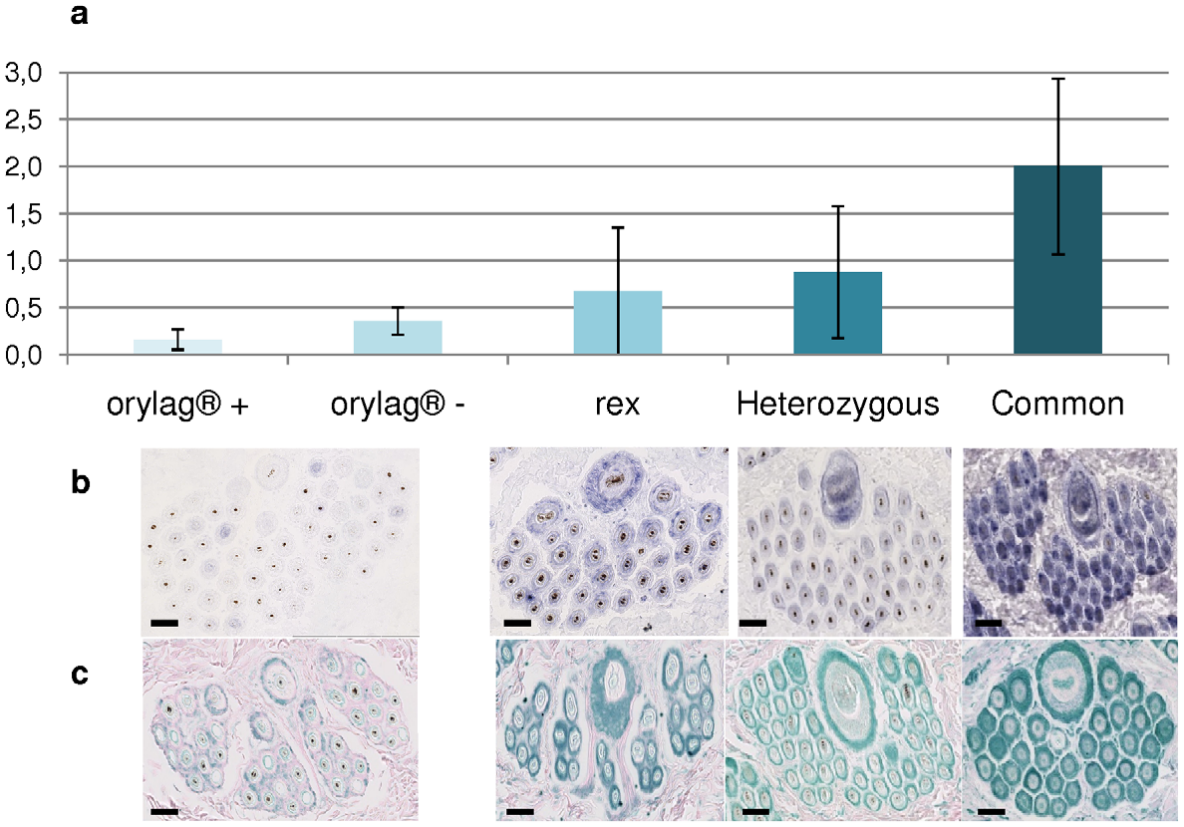
*LIPH* expression in adult *orylag®+, orylag®−,* rex, heterozygous and normal rabbit skins. (Bars = 20 µm). (A) Q-PCR expression of *LIPH* mRNA. The Y axis represents the relative expression level of *LIPH*. (B) *In situ* hybridization. (C) Immunohistochemistry.

In adults, we compared by qPCR the three genotypes at the *LIPH* locus as well as extreme phenotypes within the *orylag®* population homozygous for the *LIPH* deletion (Figure 2). *LIPH* mRNA was detected by qPCR in homozygous and heterozygous wild type adults, rex and *orylag®* skin. The expression level of *LIPH* mRNA was found to be closely related to the genotype. The skin of rex rabbits expressed about three times less *LIPH* mRNA compared to that in the skin of normal animals (H = 8.2; p = 0.004). The skin of average *orylag®* rabbits expressed about seven times less *LIPH* mRNA compared to that of the skin of normal animals (H = 16.8; p = 4.10^−5^). In addition, within the *orylag®* phenotype, the rabbits with less guard hairs (*orylag®+*) had significantly less *LIPH* mRNA than those with more guard hairs (*orylag®*−) (H = 5.1; p = 0.02) (Figure 2A). The level in the heterozygous genotype is intermediary and significantly different to that of *orylag®+* (H = 6.4; p = 0.01) and normal rabbits (H = 4.3; p = 0.03) with whom they were mated. The *LIPH* mRNA (Figure 2C) and protein (Figure 2C) were detected by *in situ* hybridization and immunohistochemistry respectively in skin sections of all genotypes and phenotypes. We observed a gradient of the *LIPH* mRNA and protein expression, which approximately agreed with those of the qPCR results, with the highest expression in normal rabbits and the lowest expression in the finest coats of *orylag®,* while the heterozygous and the rex were intermediate. It was not possible to differentiate between *orylag®+* and *orylag®−* since the signal was very weak in both cases. In normal coat rabbits, the *LIPH* mRNA and protein were detected in the IRS (inner root sheath) and ORS (outer root sheath) of the three hair follicle classes. For all genotypes and phenotypes, the m*PA-PLA1* is less expressed in the IRS compared to the ORS. In *orylag®*, *LIPH* was only clearly detected in the ORS.

### *LIPH* expression during hair follicle cycle

**Figure 3:**
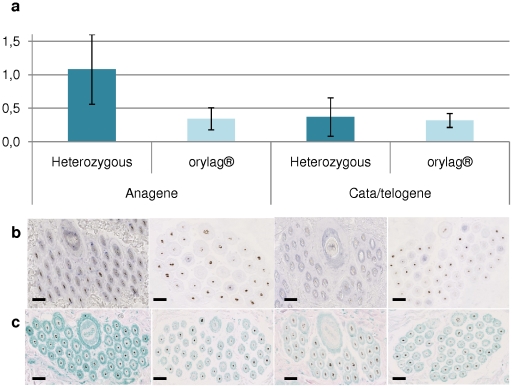
*LIPH* expression in *orylag®* and heterozygous skin of adult synchronized rabbits at the anagen and catagen/telogen follicle cycle stages. (Bars = 20 µm). (A) Q-PCR expression of *LIPH* mRNA. The Y axis represents the relative expression level of *LIPH*. (B) *In situ* hybridization. (C) Immunohistochemistry.

There was no difference for *LIPH* between the two cycle stages in the *orylag®* (H = 0.05; p = 0.8), on the contrary to heterozygous rabbits that present a significant higher expression level (more than twice) of *LIPH* mRNA at the anagen stage compared to the catagen/telogen stage (H = 5.2; p = 0.02). Whatever the stage, mRNA in *orylag®* was much less expressed than in heterozygous animals (Figure 3A). There was no significant difference between *LIPH* levels at the catagen/telogen stage in normal rabbits and both values for *orylag®* (H = 0.02; p = 0.9). *In situ* hybridization indicated that the *LIPH* mRNA was weakly expressed in the *orylag®* and at the same low level in both stages. In heterozygous animals, the level of expression was much higher in the anagen compared to the catagen/telogen stage (Figure 3B). Similarly, mPA-PLA1α was weakly expressed in the *orylag®* and there was no difference of expression between the two stages. On the contrary, the mPA-PLA1α was clearly more expressed in the anagen stage compared to the catagen/telogen stage in heterozygous animals. In these rabbits, the expression was of the same order of magnitude at the catagen/telogen stage than that of *orylag®* at either stages (Figure 3C). These results show that *LIPH* mRNA and protein levels approximately agree with those of qPCR results.

### mPA-PLA1α enzymatic activity in cell cultures

The activity of the 1362delA *LIPH* mutation on lipid metabolism was assessed by an *in vitro* assay as described by Pasternack [17] using PED-A1 as a substrate. A highly significant difference between wild-type mPA-PLA1α and 1362delA mPA-PLA1α activities was observed (H = 8.5; p = 0.003) (Table 1). At 10 minutes, the 1362delA mPA-PLA1α activity was that of the background while the wild-type mPA-PLA1α already started converting the substrate by its phospholipase activity. After 30 minutes, the wild-type mPA-PLA1α activity was 1.5 higher than that of 1362delA mPA-PLA1α, a difference conserved after one hour of reaction.

**Table 1:** Lipase activity of wild-type *LIPH* and 1362delA *LIPH* mutant constructs.

Time (min)	1362delA *LIPH*	Wild-type *LIPH*	Ratio
10	0,85	1,49	1,8
30	1,22	1,88	1,5
60	1,35	2,01	1,5

Values obtained are shown as a percentage of that observed in cells transfected with the empty vector (pcDNA3.1+, control).

## Discussion

We first provide evidence showing that the 1362delA *LIPH* mutation [3] does not alter the histological structure of rabbit skin. Indeed, no gross abnormalities were observed after staining of skin cross sections, neither at the three fetal stages, nor in adults. Hair follicle structures were similar between *orylag®* and normal rabbits at all development stages. Likewise, no differences of hair follicle group density could be evidenced. These data suggest that *LIPH* does not play a major role in hair follicle formation and development. We then show that *LIPH* is expressed in three hair follicle types, mainly in the ORS and to a lesser extent in the IRS. These findings raise the question of how this *LIPH* mutation affects mainly primary hair follicles producing guard hair. In orylag® rabbits, *LIPH* was only clearly detected in the ORS with no detectable signal in the IRS. This could mean that the orylag® selection suppressed the *LIPH* transcription in the IRS. However, due to the weaker *LIPH* signal in rex and *orylag®* follicles, we cannot rule out that we are at the detection limit of the method.

Expression of the *LIPH* gene was also monitored by qPCR on depilated skin to compare the different genotypes at different development stages. We provide evidence that *LIPH* is regulated during hair follicle cycles. Expression levels decrease by a factor 3 in normal rabbits between anagen and catagen/telogen stages. Interestingly, the *LIPH* mRNA expression does not seem to be regulated during development in the *orylag®* strain and the expression level appears similar to that of normal rabbits at the catagen/telogen stage. This suggests that *LIPH* may be required for the migration and/or differentiation of keratinocytes into distinct epithelial hair lineages during the anagen phase, when hairs are growing [15].

*Orylag®* and rex rabbits exhibited a marked decrease of *LIPH* mRNA level compared to control rabbits at both fetal and adult stages, in agreement with *in situ* hybridization. Thus, the *LIPH* mutation induces a reduced expression of both mRNA and protein. One possible explanation could be the nonsense mediated decay (NMD) mechanism [18] but the *LIPH* 1362delA characteristics are not in agreement with NMD or STAU1-mediated decay [19] criteria. We also explored the possible involvement of regulatory elements. Interestingly, the 1362delA mutation leads to a perfect match with the seed sequence of hsa-miR-518c*, also described in mice (mmu-miR-1904) and primates (ppt-miR1031a and b). This miRNA was shown to be down-regulated in psoriatic skin [20]. This miRNA could play a major role in the decrease of *LIPH* mRNA expression in rex rabbits if expressed in rabbit skin. We cannot exclude that the 1362delA mutation could also affect the secondary mRNA structure leading to its degradation.

Interestingly, *orylag®* individuals conserve a variability of *LIPH* RNA levels, discriminating between best *orylag®* with a perfect fur (down exclusively, *orylag®+*) and low quality *orylag®* furs(*orylag®−*). These findings suggest that some particular features of the rex allele and/or some other regulatory genes may have been selected in *orylag®* animals. We thus investigated two regions located 500 bp and 3 kb downstream of the transcription start site predicted to contain binding sites for transcription factors involved in hair follicle development and maintenance [21]–[23]. No polymorphisms provoking changes in these regions could be identified among normal, rex and *orylag®* (data not shown). Other genes with more tenuous action on the hair growth process may also have been selected during the improvement of the rex trait to obtain the *orylag®* phenotype.

The decrease of the mutant mPA-PLA1α activity could be the result of the deletion, directly reducing the intrinsic activity of the protein or that the normally active protein does not localize at the right place at the cell membrane. Confocal microscopy should be able to clarify this point.

Here, we show that the rex mutation, not located in the lipase domain of mPA-PLA1α, does not totally disrupt the lipase activity. It thus seems that the remaining activity is sufficient to produce down hair and nanified coarse hairs.

Another noticeable point is that mPA-PLA1α, belonging to the pancreatic lipase family, needs a colipase to open and activate its catalytic domain. The C-terminal region contains a motif which, when recognized by the colipase, allows it to match the lid domain. *In silico* protein structure prediction of both normal and mutant mPA-PLA1α rabbit protein suggests that the mutant protein is unable to match with the colipase. This could explain the decrease in the lipase activity.

The 1362delA mutation could also affect the tertiary structure of the mPA-PLA1α and interfere with its cell membrane expected localization. The modeling indicates that the rex protein presents a destabilization of the C-terminal domain in comparison with the normal protein. The mutant mPA-PLA1α could thus present a default of localization, hampering normal activity.

In contrast to the pathological defects reported in humans, the situation of rex and *orylag®* rabbits is considered more as an advantage than as a defect. This is due to the fact that the deletion reduces but does not abolish the hair growth. Our rabbit mutant and normal phenotypes represent a most suitable model to better understand the function of *LIPH* in the hair growth and development process and should stimulate new investigative areas for human applications. Further investigations are needed to explore the mode of action of this gene in such a complex, but so instructive, mechanism of hair growth cycling.

## Materials and Methods

### Animals and tissue collection

Mating plans were achieved in order to obtain rabbit fetuses at 19, 25 and 29 days postcoïtum. One normal and one *orylag®* female were used for each stage leading to the collection of 5 to 6 fetuses per female. We pooled the fetus tissues of the same litter. Adult rabbits 11–14 weeks old were selected to ensure obtaining fully developed hair follicle groups. Skin biopsies were made on 7 *orylag®+,* 7 *orylag®−*, 10 rex, 10 normal (INRA2066 strain) and 10 heterozygous for 1362delA *LIPH* rabbits. We produced adult rabbits with hair cycles in phase for the growing anagen and the resting catagen/telogen by plucking skin areas of 20 cm^2^. Skin specimens were immediately snap frozen in liquid nitrogen and stored at −80°C.

In France, no mandatory ethical committee approval is yet necessary to conduct experiments on the animals of this study.

### RNA preparation and RT-qPCR analysis

Skin samples were processed for total RNA extraction using RNA Now reagent (Ozyme) as described by the manufacturer. Reverse transcriptions (RT) were performed on 5 µg of total RNA using the Superscript First Strand Synthesis System (Invitrogen) according to the manufacturer's instructions. Quantitative PCR were performed on RT products using the Mastercycler Realplex (Eppendorf). Reaction conditions consisted of 15 min at 95°C (1 cycle), 15 s at 95°C, 30 s at 60°C and 30 s at 72°C(40 cycles) and final 5 min at 72°C with primers (5 µM) using Absolute QPCR Sybr-Green (Thermo Scientific). Primers are presented in Table 2. The relative abundance of each gene was determined by the formula 2 ΔCt [24]. The ΔCt value corresponds to the Ct of each gene after normalization with the GAPDH housekeeping gene.

**Table 2:** Primer pairs used for PCR amplification, qPCR and ISH.

Primers name	Forward	Reverse	Annealing temperature (°C)
qPCR *LIPH*	TCCACGGGAGCTGTAATAGG	TGTTTCCACATTTTCCGTCA	60
qPCR *GAPDH*	GCCCAGAACATCATCCCTGC	CGTATTTGGCAGCTTTCTCC	60
ISH-*LIPH*	GACCCTCCAATGACTAAAGCA	TGCAAGCAGACTCACTTGATG	58
cDNA *LIPH*	AGGGGAAACCTTAAGCTCCGCGCG	CTTTTTGTGCCTCGAGGAAAAGTAA	55

### Histology

Rabbit skins were fixed 48 h in formol 10% at room temperature, dehydrated through successive baths of ethanol (70%, 2×5 h; 80%, 2×5 h; 95%, 5 h and 100%, 6 h) and xylene substitute (Ultraclear®, 2×6 h). Then, they were embedded in three successive baths of paraplast (60°C, 3×5 h). Paraffin sections (5 µm) were cut at room temperature and mounted on superfrost® plus slides, air dried overnight at 45°C and stored at room temperature until use for the IHC and ISH treatment. Skin sections on slides were stained with ROAN solution and then dipped into nuclear red for 7 min and rinced before being dipped into Orange G for 4 min and in Aniline blue for 3 min, dehydrated and mounted.

### *In situ* hybridization

A 213 pb normal rabbit *LIPH* cDNA was amplified with “ISH primers” (Table 2) for *in vitro* RNA probe synthesis and cloned into the pGEM-T vector system (Promega A3600). ISH riboprobes were synthetized with sp6 and T7 promoters. The transcription mixture included 1 µg of linearized plasmid (from M13 amplification), dNTP mix with DIG-UTP (Roche, DIG RNA labelling mix), RNase Inhibitor (Roche), and sp6 or T7 RNA polymerase (Roche). Tissues on slides were deparaffined and rehydrated through successive baths of xylene and ethanol and then in PBS-Tween 0.1% before being fixed with PFA 4% for 20 minutes and equilibrated in SSC5X for 15 min. Prehybridizations were performed with hybridization buffer pre-warmed at 85°C (50% formamide, 1× salt buffer, 10% dextran sulfate, 1 mg/mL yeast RNA, 1× Denhardt) during 1 hour, at 65°C in a dry oven. Hybridization was performed overnight at 65°C in a dry oven, with *LIPH* riboprobes diluted at 1/200 with hybridization buffer. Slides were rinsed through successive baths of SSC and blocked 1 h30 in MABT-Normal Goat Serum 20% at room temperature. Then, hybridization with the alkaline phosphatase-coupled anti-digoxigenin antibody (Roche), 1/2000 diluted on MABT-Normal Goat Serum 2%, was performed overnight at +4°C. Revelation was performed with the substrate of the alkaline phosphatase BMPurple (Roche) for 72 h (4–5 days for fetuses, *data not shown*). Slides were washed in PBS and PFA 4%, and mounted with an aqueous mounting media (abcys, H-5501).

### Immunohistochemistry

Tissues on slides were deparaffined and rehydrated through successive baths. The protocol involved a polyclonal antibody produced in the chicken against the rabbit *LIPH* peptide YHQVSLLARFNQDLDKVAE (Proteogenix®, 1/200), a horseradish peroxidase secondary antibody (HRP goat polyclonal to chicken IgY, Abcam) and Histogreen (Abcys) as chromogens for the peroxidase activity. Fetal slides were led to saturation in order to get a sufficient signal.

### Enzymatic activity

Each plasmid was transfected into CHO cells as described previously [25]. *LIPH* wild-type and *LIPH* 1362delA were amplified with the “cDNA *LIPH*” primers (Table 2). After maxiprep (Nucleobond® PC100, Macherey-Nagel), PCR products and empty pcDNA3.1+ vector (Invitrogen) were double digested by KpnI and XhoI (Biolabs). The two *LIPH* constructs were transformed into pcDNA3.1 plasmid and amplified by maxiprep. CHO cells (Invitrogen) from passages 2–3 were then transiently transfected with the two *LIPH* constructs or with the empty vector. Cells were seeded into a 96-well plate (BD Biosciences, Franklin Lakes, NJ). At day 2 after transfection, the medium was replaced by HBSS (Hank balanced salt solution) containing 0.5 mM PED-A1 (Invitrogen). mPA-PLA1α enzyme activity was monitored using the PED-A1 substrate quenching system (Invitrogen). The phospholipase activity was monitored every minute for 1 hour at 37°C using an epi-fluorescence microscope (excitation filter 485 nm, emission filter 540 nm).

## References

[1] Rougeot J, Thebault R-G (1989).

[2] Castle W-E, Nachtsheim H (1933). Linkage interrelations of three genes for rex (short) coat in the rabbit,. Proceedings of the national Academy of Sciences USA.

[3] Diribarne M, Mata X, Chantry-Darmon C, Vaiman A, Auvinet G (2011). A deletion in exon 9 of the LIPH gene is responsible for the rex hair coat phenotype in rabbits (Oryctolagus cuniculus).. PLoS One.

[4] Takahashi T, Kamimura A, Hamazono-Matsuoka T, Honda S (2003). Phosphatidic acid has a potential to promote hair growth in vitro and in vivo, and activates mitogen-activated protein kinase/extracellular signal-regulated kinase kinase in hair epithelial cells.. J Invest Dermatol.

[5] Wen XY, Bryce DM, Breitman ML (1998). Characterization of lpd (lipid defect): a novel mutation on mouse chromosome 16 associated with a defect in triglyceride metabolism.. Hum Mol Genet.

[6] Kazantseva A, Goltsov A, Zinchenko R, Grigorenko AP, Abrukova AV (2006). Human hair growth deficiency is linked to a genetic defect in the phospholipase gene LIPH.. Science.

[7] Ali G, Chishti MS, Raza SI, John P, Ahmad W (2007). A mutation in the lipase H (LIPH) gene underlie autosomal recessive hypotrichosis.. Hum Genet.

[8] Jelani M, Wasif N, Ali G, Chishti M, Ahmad W (2008). A novel deletion mutation in LIPH gene causes autosomal recessive hypotrichosis (LAH2).. Clin Genet.

[9] Naz G, Khan B, Ali G, Azeem Z, Wali A (2009). Novel missense mutations in lipase H (LIPH) gene causing autosomal recessive hypotrichosis (LAH2).. J Dermatol Sci.

[10] Petukhova L, Shimomura Y, Wajid M, Gorroochurn P, Hodge SE (2009). The effect of inbreeding on the distribution of compound heterozygotes: a lesson from Lipase H mutations in autosomal recessive woolly hair/hypotrichosis.. Hum Hered.

[11] Pasternack SM, Murugusundram S, Eigelshoven S, Müller M, Kruse R (2009). Novel mutations in the P2RY5 gene in one Turkish and two Indian patients presenting with hypotrichosis and woolly hair.. Arch Dermatol Res.

[12] Shimomura Y, Garzon MC, Kristal L, Shapiro L, Christiano AM (2009). Autosomal recessive woolly hair with hypotrichosis caused by a novel homozygous mutation in the P2RY5 gene.. Exp Dermatol.

[13] Horev L, Tosti A, Rosen I, Hershko K, Vincenzi C (2009). Mutations in lipase H cause autosomal recessive hypotrichosis simplex with woolly hair.. J Am Acad Dermatol.

[14] Shinkuma S, Akiyama M, Inoue A, Aoki J, Natsuga K (2010). Prevalent LIPH founder mutations lead to loss of P2Y5 activation ability of PA-PLA1alpha in autosomal recessive hypotrichosis.. Hum Mutat.

[15] Sonoda H, Aoki J, Hiramatsu T, Ishida M, Bandoh K (2002). A novel phosphatidic acid-selective phospholipase A1 that produces lysophosphatidic acid.. J Biol Chem.

[16] Hiramatsu T, Sonoda H, Takanezawa Y, Morikawa R, Ishida M (2003). Biochemical and molecular characterization of two phosphatidic acid-selective phospholipase A1s, mPA-PLA1alpha and mPA-PLA1beta.. J Biol Chem.

[17] Nahum S, Pasternack SM, Pforr J, Indelman M, Wollnik B (2009). A large duplication in LIPH underlies autosomal recessive hypotrichosis simplex in four Middle Eastern families.. Arch Dermatol Res.

[18] Kuzmiak HA, Maquat LE (2006). Applying nonsense-mediated mRNA decay research to the clinic: progress and challenges.. Trends Mol Med.

[19] Hosoda N, Kim YK, Lejeune F, Maquat LE (2005). CBP80 promotes interaction of Upf1 with Upf2 during nonsense-mediated mRNA decay in mammalian cells.. Nat Struct Mol Biol.

[20] Zibert JR, Løvendorf MB, Litman T, Olsen J, Kaczkowski B (2010). MicroRNAs and potential target interactions in psoriasis.. J Dermatol Sci.

[21] Xie Z, Chang S, Oda Y, Bikle DD (2006). Hairless suppresses vitamin D receptor transactivation in human keratinocytes.. Endocrinology.

[22] Teichert A, Elalieh H, Bikle D (2010). Disruption of the hedgehog signaling pathway contributes to the hair follicle cycling deficiency in Vdr knockout mice.. J Cell Physiol.

[23] Sánchez-Martínez R, Zambrano A, Castillo AI, Aranda A (2008). Vitamin D-dependent recruitment of corepressors to vitamin D/retinoid X receptor heterodimers.. Mol Cell Biol.

[24] Livak KJ, Schmittgen TD (2001). Analysis of relative gene expression data using real-time quantitative PCR and the 2(-Delta Delta C(T)) Method.. Methods.

[25] Vilette D, Andreoletti O, Archer F, Madelaine MF, Vilotte JL (2001). Ex vivo propagation of infectious sheep scrapie agent in heterologous epithelial cells expressing ovine prion protein.. Proc Natl Acad Sci U S A.

